# Aesculetin Attenuates Alveolar Injury and Fibrosis Induced by Close Contact of Alveolar Epithelial Cells with Blood-Derived Macrophages via IL-8 Signaling

**DOI:** 10.3390/ijms21155518

**Published:** 2020-08-01

**Authors:** Su Yeon Oh, Yun-Ho Kim, Min-Kyung Kang, Eun-Jung Lee, Dong Yeon Kim, Hyeongjoo Oh, Soo-Il Kim, Woojin Na, Young-Hee Kang

**Affiliations:** Department of Food Science and Nutrition and The Korean Institute of Nutrition, Hallym University, Chuncheon 24252, Korea; suy0411@naver.com (S.Y.O.); royalskim@hallym.ac.kr (Y.-H.K.); mitholy@hallym.ac.kr (M.-K.K.); reydmswjd@naver.com (E.-J.L.); ehddus3290@naver.com (D.Y.K.); ohhyeongju@gmail.com (H.O.); ky4850@naver.com (S.-I.K.); nsm0729@hanmail.net (W.N.)

**Keywords:** aesculetin, alveolar cells, inflammation, interleukin-8, macrophages, polyhexamethylene guanidine, pulmonary fibrosis

## Abstract

Pulmonary fibrosis is a disease in which lung tissues become fibrous and thereby causes severe respiratory disturbances. Various stimuli induce infiltration of macrophages to the respiratory tract, secreting inflammatory cytokines, which subsequently leads to the development of pulmonary fibrosis. Aesculetin, a major component of the sancho tree and chicory, is known to biologically have antioxidant and anti-inflammatory effects. Human alveolar epithelial A549 cells were cultured for 24 h in conditioned media of THP-1 monocyte-derived macrophages (mCM) with 1–20 μM aesculetin. Micromolar aesculetin attenuated the cytotoxicity of mCM containing inflammatory tumor necrosis factor-α (TNF)-α and interleukin (IL)-8 as major cytokines. Aesculetin inhibited alveolar epithelial induction of the mesenchymal markers in mCM-exposed/IL-8-loaded A549 cells (≈47–51% inhibition), while epithelial markers were induced in aesculetin-treated cells subject to mCM/IL-8 (≈1.5–2.3-fold induction). Aesculetin added to mCM-stimulated A549 cells abrogated the collagen production and alveolar epithelial CXC-chemokine receptor 2 (CXCR2) induction. The production of matrix metalloproteinase (MMP) proteins in mCM-loaded A549 cells was reduced by aesculetin (≈52% reduction), in parallel with its increase in tissue inhibitor of metalloproteinases (TIMP) proteins (≈1.8-fold increase). In addition, aesculetin enhanced epithelial induction of tight junction proteins in mCM-/IL-8-exposed cells (≈2.3–2.5-fold induction). The inhalation of polyhexamethylene guanidine (PHMG) in mice accompanied neutrophil predominance in bronchoalveolar lavage fluid (BALF) and macrophage infiltration in alveoli, which was inhibited by orally administrating aesculetin to mice. Treating aesculetin to mice alleviated PHMG-induced IL-8-mediated subepithelial fibrosis and airway barrier disruption. Taken together, aesculetin may antagonize pulmonary fibrosis and alveolar epithelial barrier disruption stimulated by the infiltration of monocyte-derived macrophages, which is typical of PHMG toxicity, involving interaction of IL-8 and CXCR2. Aesculetin maybe a promising agent counteracting macrophage-mediated inflammation-associated pulmonary disorders.

## 1. Introduction

Lung inflammation is usually induced by pathogens or by exposure to toxins, pollutants, irritants, and allergens [1,2]. During lung inflammation diverse inflammatory cells are activated, and then these cells release cytokines and mediators to modify actions of other inflammatory cells in paracrine manners [3,4]. Orchestration of these cells and inflammatory mediators leads to progression of lung inflammation. Acute lung inflammation that drives alveolar hemorrhage is primarily induced by the release of inflammatory cytokines and chemokines [4,5]. Released cytokines and chemokines stimulate an influx of neutrophils into the airways [6]. In fact, neutrophils are the predominant immune infiltrate into the lung in patients with active pulmonary fibrosis [3,7]. There are acute episodes or exacerbations in asthma and chronic obstructive pulmonary disease (COPD) characteristic of chronic inflammation of the respiratory tract [8]. In patients with COPD, there is a typical pattern of inflammation with increased numbers of neutrophils and macrophages in the airway lumen [8]. The increase in neutrophils results in an increase in the production of CXC-chemokines, such as CXC-chemokine ligand 1 (CXCL1) and CXCL8 (IL-8), that bind to CXC-chemokine receptor 2 (CXCR2) predominantly expressed in neutrophils. The inflammation that occurs in asthma is often described as eosinophilic, and eosinophil chemotactic factors, such as CC-chemokine ligand 11 (eotaxin-1) and related CC-chemokines, are mainly released by airway epithelial cells. On the other hand, the number of macrophages increased in the lungs of patients with asthma and COPD, in which many more macrophages are infiltrated in COPD than in asthma. To date, blood monocytes, as well as lung resident macrophages, have been described as main orchestrators of termination and resolution of inflammation in the lungs of patients with COPD [9,10].

A growing body of evidence supports a role for both alveolar macrophages and interstitial resident macrophages as initiators of parenchymal repair processes in the pathogenesis of lung fibrosis [11]. In addition, neutrophils have been shown to be the predominant immune infiltrate in the lung in patients with active lung fibrosis [7,12]. Fibrotic myofibroblasts are shown to be responsible for repairing extracellular matrix (ECM) in lung tissues [13]. However, the pathogenesis of pulmonary fibrosis is not well understood. Indeed, the crosstalk among alveolar epithelial cells, pulmonary macrophages, neutrophils or T-lymphocytes amplify the inflammatory responses and initiate cellular repair mechanisms [10,14]. Pulmonary fibrosis arises as a consequence of chronic inflammation and an aberrant wound-healing following repetitive alveolar epithelial injury [10,15,16]. The injured alveolar epithelium produces pro-inflammatory and pro-fibrotic factors in a state of senescence-associated secretory phenotype [17]. These immune cells perpetuate epithelial cell apoptosis and proliferation by release of pro-fibrotic growth factors, and continuous deposition of ECM thickens the basement membrane, resulting in adverse impacts on alveolar epithelial cell function [17].

**Figure 1:**
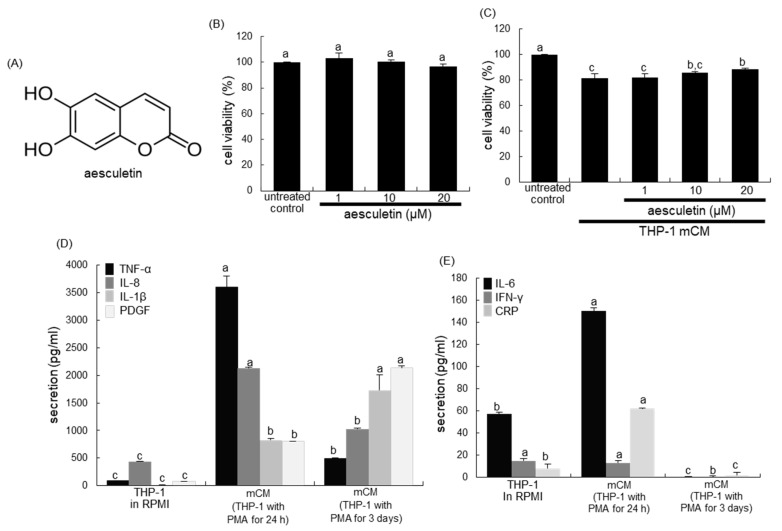
Chemical structure (**A**) and A549 cell toxicity of aesculetin (**B**) in THP-1 monocyte-derived macrophage conditioned media (mCM, **C**). Alveolar epithelial A549 cells were incubated for 24 h with 1–20 μM aesculetin (**B**), or cells were cultured with 1–20 μM aesculetin for 24 h in mCM (**C**). Cell viability was measured by 3-(4,5-dimetylthiazol-yl)-diphenyltetrazolium bromide assay and expressed as percent cell survival relative to untreated controls (cell viability = 100%, mean ± SEM, *n* = 5). THP-1 monocytes were exposed to phorbol myristate acetate (PMA) for 1 day or 3 days and media were collected (**D**,**E**). Release of tumor necrosis factor-α (TNF-α), interleukin (IL)-8, IL-1β, platelet-derived growth factor (PDGF), IL-6, interferon-γ (IFN-γ) and C-reactive protein (CRP) in mCM obtained from THP-1 monocytes exposed to 50 ng/mL PMA for 1 day or 3 days was detected by using ELISA kits. Respective values in bar graphs not sharing a common letter indicate significant different at *p* < 0.05.

Several studies have demonstrated that polyphenolics and other active constituents present in plants play a significant role in repairing the pulmonary damages [18,19,20]. Aesculetin (Figure 1A), a coumarin derivative present in various natural plants, has anti-inflammatory property and anti-tumor activity [21,22,23]. A recent study shows that aesculetin displays anti-inflammatory and protective effects against lipopolysaccharide (LPS)-induced acute lung injury via modulation of inflammatory pathways [24]. However, little is known about the therapeutic effects of aesculetin on blood-derived immune cell infiltrate-induced pulmonary fibrosis and disruption of the epithelial junction barrier. Based on the literature evidence that the blood-derived immune cells of neutrophils and macrophages affect alveolar epithelial function, the current study elucidated that aesculetin abrogated aberrant alterations of the phenotypes of alveolar epithelial cells exposed to monocyte-derived macrophage conditioned media (mCM) and neutrophil chemotactic interleukin (IL)-8. Furthermore, this study examined whether aesculetin ameliorated pulmonary fibrosis of mice exposed to polyhexamethylene guanidine (PHMG) used as a biocidal disinfectant, through diminishing IL-8-mediated macrophage infiltration into the lung. In fact, the pulmonary toxicity of PHMG was revealed due to an outbreak of severe lung disease in South Korea following frequent use as a humidifier disinfectant [25]. Several studies have reported the pathogenesis of PHMG-induced pulmonary inflammation and fibrosis [25,26,27]. 

## 2. Results

### 2.1. Release of Inflammatory Cytokines/CRP/PDGF of Monocyte-Derived Macrophages

This study attempted to investigate that blood-derived macrophages affected the phenotypic function of alveolar epithelial cells. When A549 cells were cultured in mCM collected from to phorbol myristate acetate (PMA)-exposed THP-1 monocytes for 24 h, the viability was significantly reduced (Figure 1C). In contrast, ≥10 μM aesculetin significantly increased their viability. 

In THP-1 monocytes treated with PMA for 24 h or 3 days, TNF-α, IL-8, IL-1β and PDGF were released in a temporal manner (Figure 1D). THP-1 macrophages promptly and highly released TNF-α and IL-8 in 24 h, but the secretion of IL-1β and PDGF was slowly enhanced in macrophages exposed to PMA for 72 h. In addition, small but significant amounts of IL-6 and CRP were secreted in PMA-treated THP-1 macrophages for 24 h (Figure 1E). Accordingly, it can be assumed that inflammatory TNF-α and IL-8 were major cytokines present in mCM.

### 2.2. Blockade of Alveolar Epithelial–Mesenchymal Transformation (EMT) by Aesculetin

**Figure 2:**
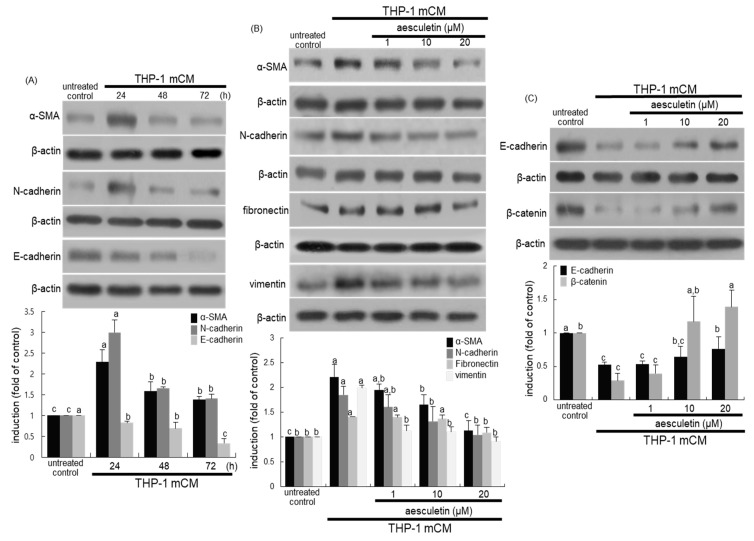
Western blot analysis showing time-course responses of epithelial–mesenchymal transformation EMT markers (**A**) and inhibitory effects of aesculetin on induction of EMT markers (**B**,**C**). Alveolar epithelial A549 cells were incubated in THP-1 monocyte-derived macrophage conditioned media (mCM) up to 72 h in the presence of 1–20 μM aesculetin. Cell lysates were subject to Western blot analysis with a primary antibody against α-smooth muscle actin (α-SMA), N-cadherin, E-cadharin, fibronectin, vimentin, and β-catenin. β-Actin protein was used as an internal control. The bar graphs (mean ± SEM, *n* = 3) represent quantitative results of the upper bands obtained from a densitometer. Means without a common letter differ, *p* < 0.05.

The current study examined whether aesculetin inhibited the transformation of alveolar epithelial cells cultured in mCM. The mesenchymal markers α-smooth muscle actin (α-SMA) and N-cadherin typical of EMT were induced in alveolar epithelial A549 cells cultured in mCM for 24 h, and thereafter their induction was abolished (Figure 2A). On the contrary, the epithelial level of E-cadherin gradually and significantly declined within 24 h (Figure 2A). When A549 cells were incubated in mCM for 24 h, enhanced alveolar expression of all the α-SMA, N-cadherin, fibronectin and vimentin was diminished by supplementing 1–20 μM aesculetin to A549 cells (Figure 2B). Additionally, micromolar aesculetin increased alveolar epithelial levels of E-cadherin and β-catenin reduced by culturing A549 cells in mCM (Figure 2C). The mCM obtained from THP-1 cells might trigger conversion of alveolar epithelial cells into a mesenchymal phenotype.

**Figure 3:**
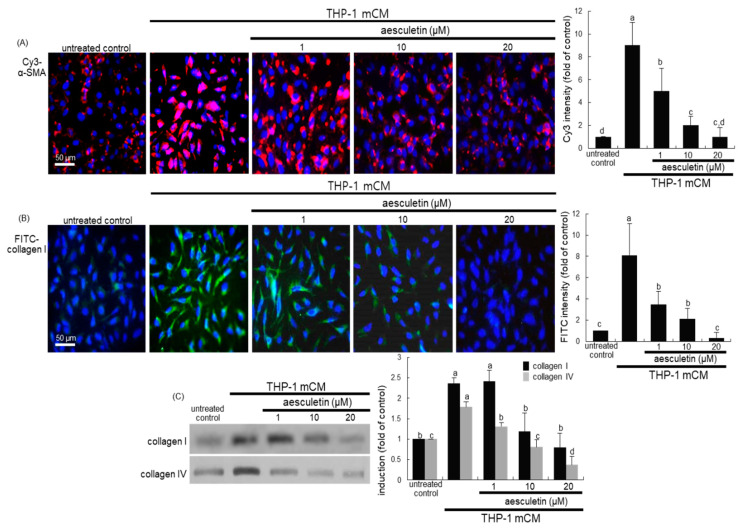
Immunocytochemical data showing inhibitory effects of aesculetin on induction of α-SMA and collagen I (**A**,**B**) and Western blot data showing inhibitory effects of aesculetin on secretion of collagen I and collagen IV (**C**). Alveolar epithelial A549 cells were incubated in THP-1 monocyte-derived macrophage conditioned media (mCM) for 24 h in the presence of 1–20 μM aesculetin. α-SMA and collagen I of A549 cells was identified as Cy3-red staining or fluorescein isothiocyanate-green staining, respectively (**A**,**B**). Nuclear counter-staining was conducted with 4′,6-diamidino-2-phenylindole (blue). Fluorescent intensity of Cy3-red staining and FITC-green staining was measured using an optical Axiomager microscope system (mean ± SEM, *n* = 3). For the secretion of collagen I and collagen IV, cell lysates were subject to Western blot analysis with a primary antibody against collagen I and collagen IV (**C**). The bar graphs (means ± SEM, *n* = 3) represent quantitative results of the left bands obtained from a densitometer. Means without a common letter differ, *p* < 0.05. Scale bars = 50 μm.

The fluorescent immunostaining images confirmed that aesculetin disturbed the alveolar EMT process, as evaluated by using a specific antibody of α-SMA or collagen type I. There was a weak staining of α-SMA in untreated cells (Figure 3A). However, in mCM-exposed A549 cells for 24 h heavy pinkish staining was observed outside blue nuclei. The pinkish staining for the α-SMA induction was substantially and dose-dependently diminished in cells treated with 1–20 μM aesculetin (Figure 3A). In addition, aesculetin inhibited the induction of cellular collagen I of alveolar epithelial cells by culturing in mCM, evidenced by fluorescein isothiocyanate (FITC)-green staining (Figure 3B). Furthermore, Western blot data revealed that the secretion of ECM components of collagen I and collagen IV was promoted in mCM-exposed A549 cells, which was markedly attenuated by adding aesculetin to cells (Figure 3C). Thus, aesculetin may suppress alveolar EMT following a direct contact of alveolar epithelial cells and blood-derived macrophages. 

### 2.3. Inhibition of mCM-Induced Alveolar Injury by Aesculetin

**Figure 4:**
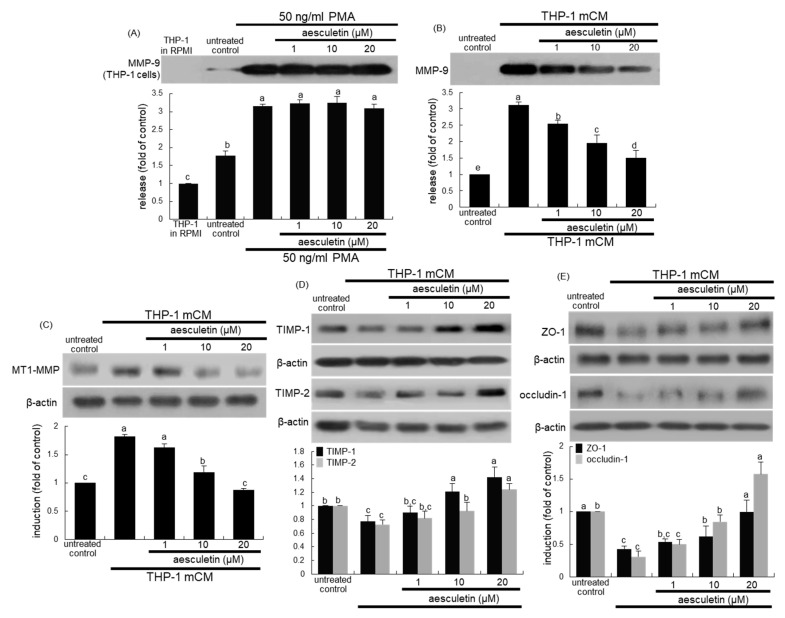
Western blot data showing effects of aesculetin on induction of alveolar epithelial tight junction markers. THP-1 monocytes were exposed to 50 ng/mL phorbol myristate acetate for 24 h in the absence presence of aesculetin (**A**). Alveolar epithelial A549 cells were treated with 1–20 μM aesculetin and exposed to THP-1 monocyte-derived conditioned media (mCM, **B**–**E**) for 24 h. After culture protocols of THP-1 monocytes and A549 cells, Western blot analysis with culture media or cell lysates was conducted with a primary antibody against matrix metalloproteinase-9 (MMP-9), MT1-MMP, tissue inhibitor of metalloproteinases (TIMP)-1, TIMP-2, ZO-1 and occludin-1. β-Actin was used as an internal control. The bar graphs (mean ± SEM, *n* = 3) for blots in the upper panels represent quantitative results obtained by densitometric analysis. Means without a common letter differ, *p* < 0.05.

It was examined whether aesculetin inhibited the matrix metalloproteinase-9 (MMP-9) secretion, as evidenced by Western blot analysis. The MMP-9 secretion was highly stimulated in THP-1 cells treated with 50 ng/mL PMA, but 1–20 μM aesculetin did not influence such secretion (Figure 4A). When A549 cells exposed to mCM containing substantial amounts of MMP-9, no further increase in MMP-9 secretion of A549 cells was observed (Figure 4B). However, 1–20 M aesculetin dampened its secretion in A549 cells cultured in mCM for 24 h in a dose-dependent manner (Figure 4B). In addition, Western blot data showed that the mCM treatment upregulated cellular expression of MT1-MMP of alveolar epithelial cells, while the addition of ≥10 μM aesculetin suppressed alveolar epithelial MT1-MMP expression (Figure 4C). On the other hand, the cellular levels of epithelial tissue inhibitor of metalloproteinases (TIMP)-1 and TIMP-2 declined in A549 cells exposed to mCM for 24 h (Figure 4D). In contrast, aesculetin dose-dependently enhanced the reduced expression of TIMP-1 and TIMP-2 in A549 cells.

This study attempted to determine that epithelial barrier damage of alveoli was associated with disruption of tight junctions amongst epithelial cells, which was ameliorated by treating aesculetin to epithelial cells. The 24 h exposure of A549 cells to mCM resulted in reduced expression of the tight junction proteins of ZO-1 and occludin-1 (Figure 4D). On the contrary, aesculetin increased epithelial induction of both ZO-1 and occludin-1. Accordingly, the mCM-stimulated secretion of MMP-9 protein may contribute to alveolar epithelial injury, leading to disruption of tight junctions amongst epithelial cells.

### 2.4. Involvement of IL-8 in Induction of EMT and Disruption of the Epithelial Barrier

**Figure 5:**
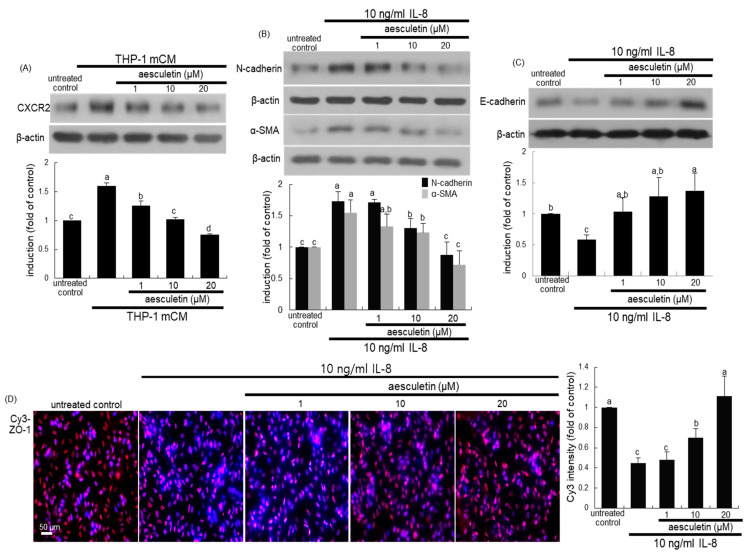
Inhibitory effects of aesculetin on CXC-chemokine receptor 2 (CXCR2) induction (**A**) by THP-1 monocyte-derived conditioned media (mCM) and induction of epithelial–mesenchymal transformation (EMT) markers by IL-8 (**B**–**D**). A549 cells were treated with 1–20 μM aesculetin in mCM or in the presence of 10 ng/mL IL-8 for 24 h. Cell lysates were subject to Western blot analysis with a primary antibody against CXCR2, N-cadherin, α-SMA, and E-cadherin (**A**–**C**). The bar graphs (mean ± SEM, *n* = 3) represent quantitative results of the upper bands obtained from a densitometer. Alveolar epithelial ZO-1 of A549 cells was identified as Cy3-red staining (**D**). Nuclear counter-staining was conducted with 4′,6-diamidino-2-phenylindole (blue). Fluorescent intensity of Cy3-red staining was measured using an optical Axiomager microscope system (mean ± SEM, *n* = 3). Respective values in bar graphs not sharing a common letter indicate significant different at *p* < 0.05. Scale bar = 50 μm.

This study elucidated that IL-8, a major cytokine in mCM, was involved in developing alveolar EMT. When alveolar epithelial A549 cells were cultured in mCM for 24 h, the IL-8 receptor CXCR2 was highly induced (Figure 5A). In contrast, the CXCR2 induction was dose-dependently attenuated in A549 cells treated with aesculetin. 

In order to reveal the involvement of IL-8 in the EMT process leading to disruption of the epithelial barrier, 10 ng/mL IL-8 was treated to alveolar epithelial cells. IL-8 promoted expression of the mesenchymal markers of N-cadherin and α-SMA (Figure 5B). However, the expression of the epithelial markers of E-cadherin was markedly suppressed in IL-8-loaded A549 cells (Figure 5C). The treatment of A549 cells with aesculetin reversed the induction of N-cadherin, α-SMA and E-cadherin. Furthermore, the immunocytochemical Cy3-red staining of ZO-1 showed that aesculetin inhibited the disruption of the epithelial barrier, which was possibly caused by IL-8 exposed to A549 cells (Figure 5D).

### 2.5. Inhibition of Inflammatory Cell Infiltration into PHMG-Exposed Airways by Aesculetin

**Figure 6:**
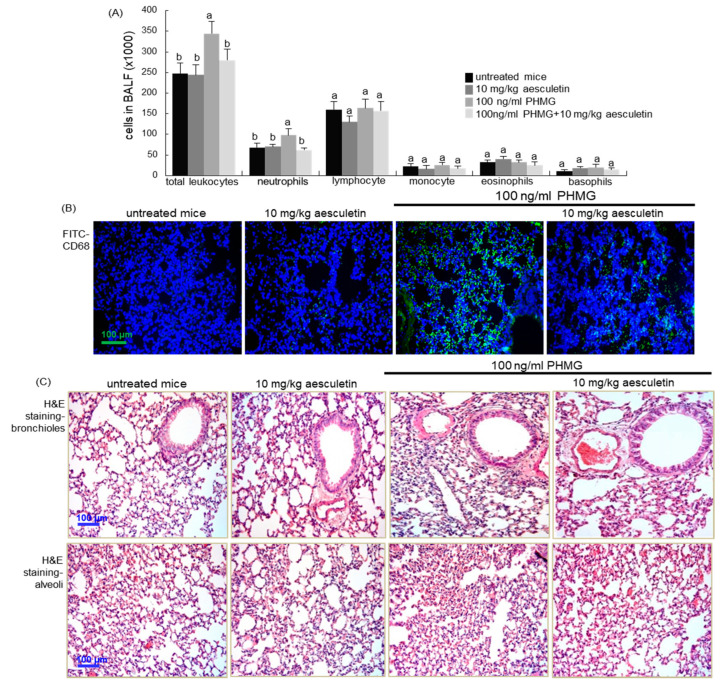
Cells in bronchoalveolar lavage fluid (BALF, **A**), macrophage infiltration (**B**) and airway fibrosis (**C**) in polyhexamethylene guanidine (PHMG) inhalation-challenged mouse lungs. PHMG-treated mice were orally administrated with 10 mg/kg aesculetin for 4 weeks. Cells in the BALF were counted using a Hemavet HV950 Multispecies Hematologic Analyzer (**A**). Respective values in bar graphs not sharing a common letter indicate significant different at *p* < 0.05. The CD68 localization was identified as FITC-green staining in mouse alveoli exposed to PHMG (**B**). Nuclear staining was done with 4′,6-diamidino-2-phenylindole (blue). For the detection of airway fibrosis, tissue sections of airways and alveoli were stained by using H&E reagent (**C**). Each photograph is representative of four mice. Scale bars = 100 μm.

This study further investigated whether inflammatory infiltration of macrophages contributed to alveolar alterations induced by PHMG inhalation in mice. When 100 ng/mL PHMG was treated to mice for 4 weeks, the counts of neutrophils in bronchoalveolar lavage fluid (BALF) increased significantly (Figure 6A). In contrast, the administration of 10 mg/kg aesculetin to PHMG-exposed mice inhibited neutrophilia in BALF. On the other hand, inflammatory macrophages were accumulated in alveoli subject to PHMG inhalation, as evidenced by FITC-green staining of the macrophage/monocyte marker CD68 (Figure 6B). In contrast, 10 mg/kg aesculetin diminished the alveolar CD68 staining. 

From the histological observation with hematoxylin and eosin (H&E) staining, the bronchioles and alveoli of mice exposed to PHMG became dense, indicating a remarkable recruitment of inflammatory cells (Figure 6C). When 10 mg/kg aesculetin was supplemented to PHMG-exposed mice, such histological changes in bronchioles and alveoli were noticeably reduced.

### 2.6. Blockade of PHMG-Induced and IL-8-Mediated Airway EMT and Fibrosis by Aesculetin

**Figure 7:**
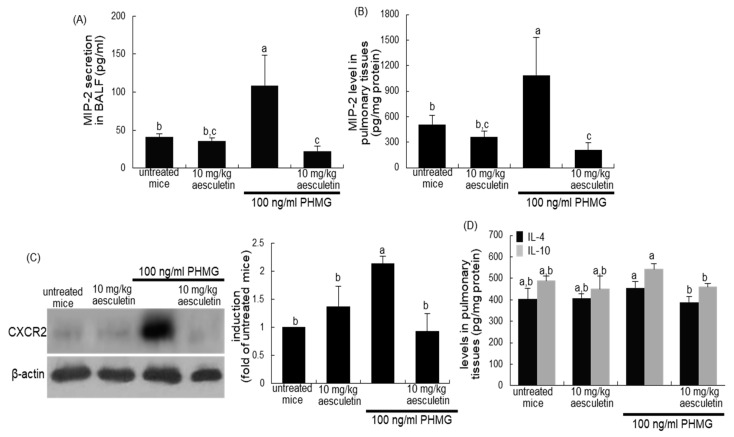
Secretion of MIP-2 in bronchoalveolar lavage fluid (BALF, **A**), and levels of MIP-2 (**B**), CXCR2 induction (**C**) and levels of IL-4 and IL-10 (**D**) in pulmonary tissues of polyhexamethylene guanidine (PHMG) inhalation-challenged mice. PHMG-treated mice were orally administrated with 10 mg/kg aesculetin for 4 weeks. The levels of MIP-2, IL-4 and IL-10 in BALF and lungs were measured using ELISA kits (**A**,**B**,**D**). Lung tissue extracts were subject to Western blot with a primary antibody against CXCR2 (**C**). β-Actin protein was used as an internal control. The bar graphs (mean ± SEM, *n* = 3) represent quantitative results of the left bands obtained from a densitometer. Respective values in bar graphs not sharing a letter indicate significant different at *p* < 0.05.

This study elucidated that IL-8 was involved in PHMG-induced structural alterations of lung tissues. Chemokines with protein sequence homology to human IL-8 have not been identified in mice [28]. The CXC chemokines of keratinocyte chemoattractant and macrophage inflammatory protein 2 (MIP-2, also known as CXCL2) are functional homologs of human IL-8 in mice. Accordingly, the MIP-2 levels in mouse lung tissue were measured. When mice were exposed to 100 ng/mL PHMG inhalation for 4 weeks, the MIP-2 secretion in BALF was elevated by 2-fold (Figure 7A). In addition, the pulmonary tissue level of MIP-2 was significantly enhanced (Figure 7B). In contrast, the administration of 10 mg/kg aesculetin reduced the MIP-2 content in BALF and lung tissues of PHMG-exposed mice (Figure 7A,B). Additionally, oral supplementation of aesculetin inhibited the pulmonary induction of CXCR2, the receptor to IL-8, in PHMG-treated mice (Figure 7C). On the other hand, the PHMG inhalation did not affect pulmonary tissue levels of IL-4 and IL-10 (Figure 7D).

**Figure 8:**
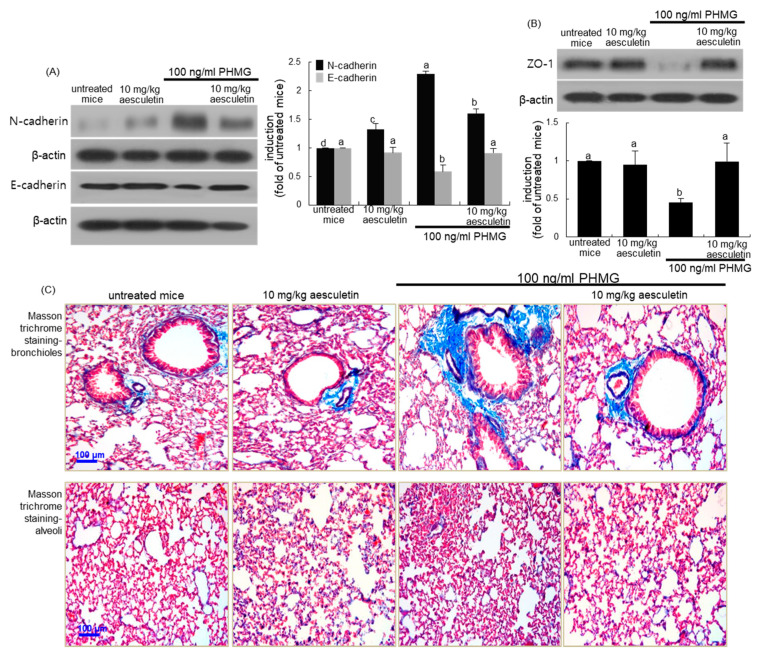
Inhibitory effects of aesculetin on markers of EMT (**A**,**C**) and tight junction (**B**) of polyhexamethylene guanidine (PHMG)-challenged mouse small airways and alveoli. Mice were orally administrated with 10 mg/kg aesculetin and exposed to 100 ng/mL PHMG for 4 weeks. Lung tissue extracts were subject to Western blot analysis with a primary antibody against N-cadherin, E-cadherin and ZO-1 (**A**,**B**). The bar graphs (mean ± SEM, *n* = 3) represent quantitative results of the left or upper bands obtained from a densitometer. Respective values in bar graphs not sharing a letter indicate significant different at *p* < 0.05. Masson trichrome staining was done in small airways and alveoli of PHMG-challenged mice (**C**). Each photograph is representative of four mice. Airway tissue sections were stained by using H&E reagent (**A**). Each photograph is representative of four mice. Scale bars = 100 μm.

This study investigated whether aesculetin ameliorated the structural alterations in lungs due to increased alveolar EMT and deposition of ECM components. The PHMG inhalation promoted pulmonary tissue level of N-cadherin, while the E-cadherin level was highly reduced (Figure 8A). In addition, the pulmonary level of ZO-1 was abolished in PHMG-loaded mice (Figure 8B). Oral treatment of PHMG-exposed mice with 10 mg/kg aesculetin reversed the induction of EMT and epithelial junction markers (Figure 8A,B). Collagen fiber deposition was notably observed (blue color) in bronchioles and alveoli of PHMG-exposed mice, as evidenced by Masson’s trichrome staining (Figure 8C). There was marked deposition of collagen fibers around the bronchi and in alveolar tissues of PHMG inhalation-challenged mice. On the contrary, the treatment with 10 mg/kg aesculetin inhibited the collagen fiber deposition, and alleviated subepithelial fibrosis in bronchioles and alveoli (Figure 8C).

## 3. Discussion

Eight major findings were extracted from this study: (1) Micromolar aesculetin attenuated the cytotoxicity of mCM collected from PMA-exposed THP-1 monocytes. (2) THP-1 monocyte-derived macrophages promptly and highly released TNF-α and IL-8 within 24 h, indicating that inflammatory TNF-α and IL-8 were major cytokines present in mCM. (3) The exposure of mCM highly induced alveolar epithelial CXCR2, which was diminished by aesculetin. (4) Aesculetin attenuated alveolar epithelial induction of the mesenchymal markers in mCM-exposed or IL-8-loaded A549 cells, while alveolar epithelial markers increased in aesculetin-treated cells subject to mCM or IL-8. (5) The addition of aesculetin to mCM-stimulated A549 cells abrogated the secretion of collagen proteins leading to alveolar epithelial fibrosis. (6) The production of MMP proteins in mCM-loaded A549 cells was diminished by aesculetin, in parallel with increased expression of TIMP proteins. (7) Aesculetin enhanced epithelial induction of tight junction proteins in mCM- or IL-8-exposed cells. (8) The PHMG inhalation in mice resulted in neutrophil predominance in BALF and macrophage infiltration in alveoli, which was inhibited by orally administrating aesculetin to PHMG-exposed mice. (9) The treatment with aesculetin alleviated PHMG-induced MIP-2-mediated subepithelial fibrosis and airway barrier disruption of mice. Taken together, aesculetin may antagonize pulmonary fibrosis and alveolar epithelial barrier disruption triggered by infiltration of monocyte-derived macrophages—which is typical of PHMG toxicity—involving interaction of MIP-2 and CXCR2. 

Numerous types of inflammatory cells are activated and release diverse cytokines, growth factors and other mediators during lung inflammation, which is usually induced by diverse pathogens or by exposure to toxins, pollutants and allergens [1,4]. During chronic inflammation, abnormal function of immune cells leads to prolongation of inflammatory cell infiltration of the lungs [1]. Chronic airway inflammation is typical in the respiratory tract of both asthma and COPD, yet there are marked differences in the inflammatory cells involved [1,8]. Inflammation in asthma only occurs in the airways, but inflammation in COPD contributes to airway remodeling and parenchymal destruction throughout the lungs [8,29]. In asthma, non-apoptotic eosinophils are prominent, and CD4+ T cells are more evident than CD8+ T cells [1,8]. However, neutrophils, macrophages, CD8+ T lymphocytes and B cells infiltrate the airway lumen of COPD, evoking chronic inflammation [1,30]. Such inflammatory process results in lung injury disordering of lung parenchymal cells and pulmonary fibrosis [31,32]. Macrophages markedly settled in the alveolar space of patients with COPD mostly due to increased recruitment of blood monocytes, and they are localized to sites of alveolar destruction [9,33]. This study investigated how monocyte-derived macrophages and their major cytokine IL-8 induced alveolar epithelial injury and fibrosis via the EMT process. In fact, the THP-1 monocyte-derived macrophages promptly released inflammatory TNF-α and IL-8 within 24 h. IL-8 is neutrophilic chemotactic cytokine. In addition, the PHMG inhalation resulted in neutrophil predominance in mouse BALF and inflammatory macrophage infiltration in alveoli. Collectively, macrophages and neutrophils may induce dysregulated inflammation and fibrosis in alveoli, leading to alveolar injury and barrier disruption. Indeed, dysregulated inflammation and macrophage polarization are critical factors in idiopathic pulmonary fibrosis and COPD [12,33,34]. 

Subepithelial fibrosis in lungs is attributed to excess deposition of ECM components in lung including collagens, resulting in overgrowth, hardening, and/or scarring of lung tissues [35]. Inflammatory monocytes and resident macrophages are key regulators of tissue repair, regeneration, and fibrosis in the lung [32,35,36]. Recent data suggest that chronic inflammation by alveolar macrophages is involved in alveolar fibrosis through several potential mechanisms [11,33,36]. There is accumulating evidence that the mechanisms driving fibrogenesis are distinct from those regulating inflammation [36]. COPD is characterized by structural changes, including epithelial disruption, smooth muscle hypertrophy/hyperplasia, airway wall fibrosis, and alveolar destruction via diverse mechanisms [37]. Myofibroblasts are localized in airways and alveoli as key cellular mediators of fibrosis in the lung, and are generated from resident mesenchymal cells and epithelial cells in EMT [38]. This study revealed that inflammatory monocyte-derived macrophages induced differentiation of alveolar epithelial cells into collagen protein-producing ones in a paracrine manner. There was a marked induction of mesenchymal markers of N-cadherin, fibronectin, vimentin and α-SMA observed in mCM-exposed alveolar epithelial cells via the EMT process, resulting in loss of the alveolar epithelial phenotype. Accordingly, differentiation of myofibroblasts via EMT was activated by paracrine signals derived from macrophages infiltrated into alveoli. Indeed, the binding of macrophage IL-8 to alveolar epithelial CXCR2 activated alveolar epithelial cells to N-cadherin- and α-SMA-positive cells, myofibroblasts, and reduced the expression of E-cadherin, an epithelial marker. 

Emerging evidence has pointed out that several pharmaceuticals available to treat COPD, including phosphodiesterase-4 inhibitors and inhaled corticosteroids, may affect the underlying process of pathologic EMT that is mechanistically progressive and irreversible [39,40]. However, the need for EMT-regulating agents with high efficacy and low cytotoxicity has led to the investigation of beneficial actions of diverse phytochemicals present in fruit and vegetables [41,42]. Bioactive natural plant compounds have been used as therapeutic alternatives through modulating anti-inflammatory, anti-fibrotic or antioxidant mechanisms of action against EMT [41]. This study found that aesculetin, a derivative of coumarin, attenuated alveolar EMT induced by blood-derived macrophage neighbors and reduced expression of collagen-producing myofibroblasts. Moreover, this natural compound inhibited the IL-8-induced alveolar EMT process through a dose-dependent reduction in alveolar CXCR2 induction. On the other hand, disruption of tight barriers of alveolar epithelial cells depends on inflammatory macrophage infiltration into alveoli [43]. Aesculetin blocked the disruption of alveolar tight junctions amongst epithelial cells. Aesculetin blunted cellular membrane type-1 matrix metalloproteinase (MT-1 MMP) induction and MMP-9 secretion, and blocked loss of tight junction proteins of ZO-1 and occludin-1 in alveolar epithelial cells in mCM- or IL-8-exposed cells. Thus, aesculetin inhibited the breakdown of alveolar epithelial barriers due to interaction between alveolar epithelial cells and monocyte-derived macrophages, ultimately maintaining alveolar epithelial barrier integrity. 

Recent studies have reported that the instillation of PHMG used as a humidifier disinfectant, induces irreversible pulmonary injury and lung fibrosis [27,44]. There were inflammatory macrophage infiltration, bronchiolo-alveolar epithelial hyperplasia and pulmonary fibrosis mainly observed in the terminal bronchioles and alveoli in the lungs of PHMG-loaded mice [27]. Pathologic examination shows typical bronchiolocentric destruction with inflammation and fibrosis and bronchial epithelial denudation with the accumulation of foamy macrophages in PHMG-exposed rats [25]. Similarly, this study found that the PHMG inhalation of mice enhanced macrophage infiltration into the alveolar space, indicating that continuous inflammatory responses by PHMG may contribute to the development of pulmonary fibrosis. Furthermore, PHMG phosphate results in damage of tight junctions and impairment of F-actin architecture integrity in human bronchial epithelial cells [44]. In the current study, PHMG inhalation stimulated the EMT process and thereby loss of tight junction proteins in lungs. A recent study shows that Akt and Notch pathways mediate PHMG phosphate, which induces EMT through the activation of Akt and Notch signaling and the induction of transcription factor ZEB2 [45]. Furthermore, the PHMG inhalation of mice enhanced MIP-2 levels in BALF and lung tissues and caused an increase in the number of inflammatory cells such as macrophages. Accordingly, IL-8/MIP-2 released from macrophages and neutrophils may be involved in the detrimental effects of PHMG on alveolar barrier integrity and lung fibrogenesis. On the other hand, several studies attempted to explore protective effects of the tyrosine kinase inhibitor nintedanib against PHMG-induced lung fibrosis in mice [46,47]. This study showed that aesculetin ameliorated pulmonary fibrosis and alveolar barrier injury induced by PHMG inhalation through disturbing macrophage infiltration and EMT via IL-8/MIP-2 signaling. One investigation shows that resveratrol, a stilbenoid polyphenol found in red wine, protects the integrity of the alveolar epithelial barrier in methamphetamine or oxidative stress-induced chronic lung injury [48]. 

Since the epithelial pigment cell population is very small in whole eyes, a disconnection between the in vitro and in vivo results may occur. Although aesculetin may serve as a therapeutic agent against alveolar inflammation and fibrosis, its dietary role in alveolar injury remains elusive. Thus, further validation is required to clarify whether an optimal intake of aesculetin may comprise a dietary treatment for alveolar injury.

## 4. Materials and Methods

### 4.1. Chemicals

RPMI media 1640 was obtained from the Sigma-Aldrich Chemical (St. Louis, MO, USA), as were all other reagents unless specifically stated otherwise. Fetal bovine serum (FBS), penicillin/streptomycin, and trypsin/EDTA were purchased from the Lonza (Walkersville, MD, USA). IL-8 protein was provided by R&D systems (Minneapolis, MN, USA). Antibodies of α-smooth muscle actin (α-SMA), E-cadherin, fibronectin, vimentin, collagen I, membrane type 1-matrix metalloproteinase (MT1-MMP), tissue inhibitor of metalloproteinase (TIMP)-1, TIMP-2, ZO-1, β-catenin and CXCR2 were purchased from the Santa Cruz Biotechnology (Dallas, TX, USA). Human N-cadherin antibody was supplied by Abcam (Cambridge, UK). Human collagen IV antibody was purchased from Bioss Antibodies (Woburn, MA, USA). Mouse monoclonal β-actin antibody was obtained from Sigma-Aldrich Chemical. Horseradish peroxidase (HRP)-conjugated goat anti-rabbit IgG and goat anti-mouse IgG were purchased from Jackson Immuno-Research Laboratories (West Grove, PA, USA). Essential fatty acid free bovine serum albumin (BSA) and skim milk were supplied by Becton Dickinson Company (Sparks, MD, USA). 4′,6-Diamidino-2-phenylindole (DAPI) was obtained from Santa Cruz Biotechnology.

Aesculetin (Sigma-Aldrich Chemical) was dissolved in dimethyl sulfoxide (DMSO) for live culture with cells; a final culture concentration of DMSO was <0.5 %.

### 4.2. Preparation of Conditioned Media

Human monocyte THP-1 cells (TIB-202, American Type Culture Collection, Manassas, VA, USA) were cultured in RPMI 1640 supplemented with 10% FBS, 2 mM glutamine, 100 U/mL penicillin, and 100 µg/mL streptomycin. THP-1 cells were sustained in 90–95% confluence at 37 °C in an atmosphere of 5% CO_2_. THP-1 monocytes were cultured with 50 ng/mL phorbol myristate acetate (PMA) for 24 h in order to differentiate them to macrophage-like cells. Subsequently, culture media were harvested for use of conditioned media (mCM).

### 4.3. Alveolar Epithelial A549 Cell Culture and Viability

Human alveolar basal epithelial A549 cells were provided by the American Type Culture Collection (CCL-185). A549 cells were cultured in RPMI 1640 supplemented with 10% FBS, 2 mM glutamine, 100 U/mL penicillin, and 100 µg/mL streptomycin. A549 cells were sustained in 90–95% confluence at 37 °C in an atmosphere of 5% CO_2_. A549 cells were treated with 1–20 μM aesculetin in mCM. In another set of experiments, A549 cells were exposed to IL-8 in the absence and presence of 1–20 μM aesculetin.

The cytotoxicity of mCM and aesculetin was determined using 3-(4,5-dimetylthiazol-yl)-diphenyltetrazolium bromide (MTT, Duchefa Biochemie, Haarlem, Netherlands) after culture of A549 cells in mCM with and without aesculetin. These cells were incubated in a fresh medium containing 1 mg/mL MTT for 3 h at 37 °C. The purple formazan product was dissolved in 0.5 mL isopropanol with gentle shaking. Absorbance of formazan was measured at λ = 570 nm using a microplate reader (Bio-Rad Model 550, Hercules, CA, USA). No alterations were observed in cell viability when 1–20 μM aesculetin was solely treated to A549 cells (Figure 1B).

### 4.4. Enzyme-Linked Immunosorbent Assay (ELISA)

The secretion of tumor necrosis factor (TNF)-α, IL-8, IL-1β, platelet-derived growth factor (PDGF) interferon (IFN)-γ, IL-6, C-reactive protein (CRP), IL-4, IL-10 and MIP-2 was examined in mCM and lung tissues by using ELISA kits (R&D System), according to the manufacturer’s instructions.

### 4.5. Western Blot Analysis

Alveolar epithelial A549 cells were seeded at 6-well plates at a density of 7 × 10^4^ cells and cultured in mCM or exposed to IL-8 in the absence and presence of 1–20 μM aesculetin. Western blot analysis was conducted using lysates and culture media of A549 cells, and lung tissue extracts prepared in 1 mM Tris-HCl (pH 6.8) lysis buffer containing 10% sodium dodecyl sulfate (SDS), 1% glycerophosphate, 0.1 mM Na_3_VO_4_, 0.5 mM NaF, and a protease inhibitor cocktail. Cell lysates containing equal amounts of proteins and equal volume of culture media were electrophoresed on 8–15% sodium dodecyl sulfate-polyacrylamide gel electrophoresis and transferred onto a nitrocellulose membrane. Blocking a nonspecific binding was performed using either 3% fatty acid-free BSA or 5% non-fat dry skim milk for 3 h. The membrane was incubated overnight at 4 °C with a specific primary antibody of α-SMA, N-cadherin, E-cadherin, fibronectin, vimentin, collagen I, collagen IV, MT1-MMP, TIMP-1, TIMP-2, ZO-1, β-catenin and CXCR2. The membrane was then applied to a secondary antibody conjugated to HRP for 1 h. Following triple washing, the target proteins were determined using the Immobilon Western Chemiluminescent HRP substrate (Millipore Corp., Billerica, MA, USA) and the Agfa medical X-ray film blue (Agfa HealthCare NV, Mortsel, Belgium). Incubation with β-actin antibody was conducted for the comparative control.

### 4.6. Immunocytochemistry

Alveolar epithelial A549 cells were grown on 24-well glass slide and incubated for 24 h in mCM in the absence or presence of 1–20 μM aesculetin. A549 cells were fixed with ice-cold 4% formaldehyde for 10 min and permeated with 0.1% Triton X-100 for 5 min. Cells were blocked using a 2% BSA for 1 h. Immunofluorescent cytochemical staining of A549 cells was performed using α-SMA or ZO-1 with Cy3-conjugated anti-mouse IgG, and collagen I with FITC-conjugated anti-mouse IgG. Nuclear staining was performed with DAPI. Each slide was mounted in a VectaMount mounting medium and images were taken using an optical Axiomager microscope system (Carl Zeiss, Oberkochen, Germany).

### 4.7. Animal Experiments

Five week-old male BALB/c mice (Hallym University Breeding Center for Laboratory Animals) were used in this study. Female mice were excluded, because of concerns that female hormone cycles would affect experiments. Mice were acclimatized for 1 week before beginning the experiments. Mice were kept on a 12 h light/12 h dark cycle at 23 ± 1 °C with 50 ± 10% relative humidity under specific pathogen-free circumstances, fed a non-purified diet, and provided with water ad libitum at the Animal Facility of Hallym University.

The present study was approved by the Hallym University Institutional Review Board and Committee on Animal Experimentation (Hallym 2017-56, approved on 14 February 2018). This study was conducted in compliance with the University’s Guidelines for the Care and Use of Laboratory Animals.

All mice were distributed among four subgroups (*n* = 6–7 for each subgroup). Mice receiving PHMG (Sigma-Aldrich Chemical) inhalation were divided into two subgroups. Mice were administered with 10 mg/kg aesculetin via oral gavage daily for 4 weeks, and 1 h after its administration mice inhaled 100 ng/mL PHMG for 30 min in a plastic chamber linked to an ultrasonic nebulizer (Clenny2 Aerosol, Medel, Italy). Control mice and aesculetin-untreated mice received PBS as the PHMG vehicle. All mice were sacrificed 24 h after the latest provocation (day 30). All mice were killed with an anesthetic dose of 0.3 g/kg avertin and 8 µg/kg tert-amyl alcohol.

The trachea was cannulated, and both lungs and airways were rinsed in 1 mL PBS for the collection of bronchoalveolar lavage fluid (BALF). The numbers of inflammatory cells were determined using a Hemavet HV950 Multispecies Hematologic Analyzer (Drew Scientific, Oxford, CT, USA). The right lungs were collected, frozen in liquid nitrogen, and kept at −80 °C until used for Western blotting with lung tissue extracts. Left lungs were preserved and fixed in 4% paraformaldehyde and then used for immunohistochemical analyses.

### 4.8. Immunohistochemical Staining

Paraffin-embedded tissue sections (5 μm thick) of alveoli were deparaffinized and hydrated in order to conduct immunofluorescent histochemical analyses. The sections were preincubated in a boiling sodium citrate buffer (10 mM sodium citrate, 0.05% Tween 20, pH 6.0) for the antigen retrieval. The tissues were blocked with 5% BSA in PBS for 1 h. A specific primary antibody against CD68 was incubated overnight with the sectioned tissues. Subsequently, the tissue sections were incubated for 1 h with fluorescein isothiocyanate-conjugated anti-rabbit IgG. For identification of nuclei, the fluorescent nucleic acid dye of DAPI was applied for 10 min. Stained tissues were mounted on slides using mounting medium (Vector Laboratories, Burlingame, CA, USA). Images of each slide were obtained with an optical microscope Axioimager system (Carl Zeiss).

### 4.9. H&E Staining

For the histological analyses of airways, small airway and alveolar specimens provided at the end of the experiments were fixed in 10% paraformaldehyde. The paraffin-embedded specimens were sectioned at 5 μm thickness, deparaffinized and stained with H&E stain for 2 min, and quickly dehydrated in 95% absolute alcohol. The H&E-stained tissue sections were examined using an optical microscope Axioimager system equipped for fluorescence illumination (Carl Zeiss). Five images were taken from each tissue section.

### 4.10. Masson Trichrome Staining

Small airway and alveolar specimens were obtained at the end of the experiments and fixed in 10% buffered formalin. The paraffin-embedded kidney tissues were sectioned at 5 μm thickness, de-paraffinized and stained with Masson trichrome stain for the light microscopic visualization of collagen fibers and muscle fibers. The stained tissue sections were examined using an optical Axiomager microscope, and three images were taken for each section.

### 4.11. Statistical Analysis

The data are presented as means ± SEM for each treatment group in the in vitro and in vivo experiments. Statistical analyses were conducted using a Statistical Analysis Systems program (version 9.4, SAS Institute, Cary, NC, USA). The number of independent experiment repeats (*n*) was provided in each figure legend. The Western blot data were converted to fold of respective untreated control (cells) or untreated control mice. Significance was determined by a one-way analysis of variance (ANOVA) and differences between any pair of means were analyzed with Duncan’s multiple-range test. For the nonparametric data, the Kruskal–Wallis H-test was used to analyze the significant difference among means. Significance was accepted at the level of *p* < 0.05.

## 5. Conclusions

The current report demonstrated that aesculetin attenuated alveolar injury during infiltration of inflammatory monocyte-derived macrophages. Such beneficial effects of aesculetin were attributed to the inhibition of IL-8 producing macrophage neighbor-induced alveolar EMT and of formation of collagen-producing myofibroblasts. In addition, aesculetin inhibited breakdown of alveolar epithelial barriers through enhancing the cellular levels of tight junction proteins. Similarly, the PHMG inhalation promoted the EMT process in parallel with macrophage infiltration and loss of epithelial tight junction proteins. Taken together, naturally occurring aesculetin ameliorated pulmonary fibrosis and alveolar barrier injury induced by PHMG inhalation through disturbing macrophage infiltration and the EMT process via IL-8.

## Abbreviations

COPD	chronic obstructive pulmonary disease
CRP	C-reactive protein
CXCR2	CXC-chemokine receptor 2
ECM	extracellular matrix
EMT	epithelial-mesenchymal transition
IFN-γ	interferon-γ
IL	interleukin
LPS	lipopolysaccharide
mCM	macrophage conditioned media
MMP-9	matrix metalloproteinase-9
MT-1 MMP	membrane type-1 matrix metalloproteinase
PDGF	platelet-derived growth factor
PHMG	polyhexamethylene guanidine
α-SMA	α-smooth muscle actin
TIMP	tissue inhibitor of metalloproteinases
TNF-α	tumor necrosis factor-α
